# A Role for MicroRNA-155 Expression in Microenvironment Associated to HPV-Induced Carcinogenesis in K14-HPV16 Transgenic Mice

**DOI:** 10.1371/journal.pone.0116868

**Published:** 2015-01-27

**Authors:** Isabel Paiva, Rui M. Gil da Costa, Joana Ribeiro, Hugo Sousa, Margarida Bastos, Ana Faustino Carlos Rocha, Paula A Oliveira, Rui Medeiros

**Affiliations:** 1 Molecular Oncology and Viral Pathology Group, CI-IPOP, Portuguese Institute of Oncology of Porto, Rua Dr. António Bernardino de Almeida, 4200–072 Porto, Portugal; 2 ICBAS, Abel Salazar Institute for the Biomedical Sciences, University of Porto, Rua de Jorge Viterbo Ferreira 228, 4050–313, Porto, Portugal; 3 LEPABE, Faculty of Engineering, University of Porto, Rua Dr. Roberto Frias s/n, 4200–465, Porto, Portugal; 4 Experimental Pathology and Therapeutics Group, CI-IPOP, Portuguese Institute of Oncology, Rua Dr. António Bernardino de Almeida, 4200–072 Porto, Portugal; 5 Virology Service, Portuguese Institute of Oncology of Porto, Rua Dr. António Bernardino de Almeida, 4200–072 Porto, Portugal; 6 Veterinary Sciences Department, University of Trás-os-Montes and Alto Douro, UTAD, Quinta de Prados, 5001–801, Vila Real, Portugal; 7 Center for the Research and Technology of Agro-Environmental and Biological Sciences (CITAB), University of Trás-os-Montes and Alto Douro, UTAD, Quinta de Prados, 5001–911, Vila Real, Portugal; 8 CEBIMED, Faculty of Health Sciences of Fernando Pessoa University, Porto, Portugal; 9 Portuguese League Against Cancer (Liga Portuguesa Contra o Cancro–Núcleo Regional do Norte), Estrada Interior da Circunvalação, n°6657, 4200–177 Porto, Portugal; University of Texas, MD Anderson Cancer Center, UNITED STATES

## Abstract

Human Papillomavirus cause a number of diseases most notably cervical cancer. K14-HPV16 transgenic mice expressing the HPV16 early genes in squamous epithelial cells provide a suitable experimental model for studying these diseases. MicroRNAs are small non-coding RNAs that play an important role in regulating gene expression and have been suggested to play an important role in cancer development. The role of miR-155 in cancer remains controversial and there is limited evidence linking this miRNA to HPV- associated diseases. We hypothesized that miR-155 expression modulates each tissue’s susceptibility to develop HPV-associated carcinogenesis. In this study, we analyzed miR-155 expression in ear and chest skin samples from 22-26 weeks old, female K14-HPV16 transgenic (HPV16+/-) and wild-type (HPV-/-) mice. Among wild-type mice the expression of miR-155 was lower in ear skin compared with chest skin (p = 0.028). In transgenic animals, in situ carcinoma was present in all ear samples whereas chest tissues only showed epidermal hyperplasia. Furthermore, in hyperplastic chest skin samples, miR-155 expression was lower than in normal chest skin (p = 0,026). These results suggest that miR-155 expression may modulate the microenvironmental susceptibility to cancer development and that high miR155 levels may be protective against the carcinogenesis induced by HPV16.

## Introduction

Human papillomaviruses (HPVs) are the most common sexually transmitted agents [1]. High-risk human papillomavirus, such as HPV16 and HPV18, are the causative agents of virtually all cases of cervical cancer and a significant proportion of other anogenital cancers, as well as some head and neck cancers [2–4].

The K14-HPV16 transgenic mouse model is particularly useful to study the development of HPV-associated squamous cells carcinomas. In this model, the expression of HPV16 early region genes (E2-E8) is driven by the cytokeratin 14 (K14) promoter/enhancer, specifically targeting epithelial basal cells [5]. Basal cells are mitotically active and thus may develop further mutations in response to a proliferative stimulus, and the expression of K14 has been shown to persist in well-differentiated squamous carcinomas [6]. The expression of the HPV oncogenes E6 and E7 induces epithelial carcinogenesis through multiple premalignant stages [7]. Accordingly, the K14-HPV16 transgenic mice develop epidermal hyperplasia that progresses to dysplasia and *in situ* carcinoma (CIS) lesions and, ultimately, to invasive cancer. This animal model simulates the multistep carcinogenesis process and thus facilitates the study of epigenetic and the genetic factors that coordinate malignant conversion and regulate neoplastic progression.

MicroRNAs (miRNAs) are small noncoding RNAs that regulate gene expression promoting inhibition of messenger RNA (mRNA) translation or its degradation [8]. In normal cells, miRNAs control several processes including proliferation, differentiation and apoptosis. These molecules are also described as key regulators in many diseases including neurological disorders, cardiovascular diseases, viral infections and cancer [9]. During carcinogenesis, some miRNAs are lost whereas others are upregulated, and in fact, previous data indicates that miRNAs may be important to distinguish subtypes of cancers, where the histological diagnosis is complex and difficult [10].

MicroRNA-155 (miR-155) plays a role in many of the above oncogenic processes. This microRNA is overexpressed in many types of cancer cells and accumulating evidence shows that miR-155 is an oncogenic microRNA. However, recent studies claim that miR-155 may display anti-oncogenic properties or promote an adequate immunological response to cancer [11,12]. MiR-155 has emerged as an essential regulator of cellular physiology, particularly important in the mammalian immune system [13–15]. Thus, a possible link between miR-155 and inflammation in cancer has been reported [16]. Moreover, miR-155 transgenic mice develop B-cell lymphoma [15], and miR-155-knock-out mice exhibit impaired immune function [17].

In HPV-associated cancers, the interplay between miR-155 and HPV genes remains elusive and poorly understood. The tumor microenvironment associated to miRNAs plays an increasingly appreciated role in cancer [18], but the microenvironment of normal tissues and its role in tumorigenesis remains poorly studied.

In this study, we aimed to evaluate the expression of miR-155 in skin samples with or without the presence of integrated HPV DNA and with different HPV-associated lesions. For this purpose, we have used K14-HPV16 transgenic mice, [19], to analyze miR-155 expression in ear and chest skin samples, evaluating its correlation with tissue microenvironment and HPV-induced carcinogenesis.

## Materials and Methods

### Transgenic mice

Generation of K14-HPV mice has been previously reported [19]. K14-HPV16 mice on a FVB/n background were generously donated by Drs. Jeffrey Arbeit and Douglas Hanahan, from the University of California, through the USA National Cancer Institute Mouse Repository. The animal experiments were approved by the University of Trás-os-Montes and Alto Douro Ethics committee, University of Trás-os-Montes and Alto Douro, UTAD, Quinta de Prados, 5001-801, Vila Real, Portugal. Adequate environmental enrichment was provided for each cage and health checks were performed daily. Before collecting the samples, all animals were anesthetized by using sodium pentobarbital, followed by intracardiac punction and exsanguination, as indicated by the Federation for Laboratory Animal Science Associations (FELASA). After one week quarantine, the animals were maintained and bred in accordance with Portuguese (Portaria 1005/92 dated October the 23^rd^) and European (EU Directive 2010/63/EU) legislation, under controlled conditions of temperature (23±2°C), light-dark cycle (12 h light/12 h dark) and relative humidity (50±10%), using hardwood bedding. A standard diet (4RF21 GLP, Mucedola, Italy) and water were provided *ad libitum*. A total of 15 female mice from consecutive litters were selected for the study.

### Genotyping of HPV16-E6 and E2

The animals were genotyped at weaning, using tail tip samples. Tissue lysis was performed by adding 300μL of MagnaPure DNA Tissue Lysis Buffer (Roche, Indianapolis, USA) and 20 μL of Proteinase K and incubating overnight (aproximately 16h) at 65°C. Nucleic acids were extracted using the High Pure Viral Nucleic Acid kit (Roche, Indianapolis, USA) following the manufacturer’s instructions. DNA quality was assessed by measuring the absorbance at 260 nm, using an UV/Visible spectrophotometer. DNA purity was assessed by the ratio of the absorbance values at 260/280 nm, using the NanoDrop spectrophotometer v3.7 (Thermo Scientific, Wilmington DE, USA). The presence of amplifiable genomic DNA was tested by polymerase chain reaction (PCR) amplification of mouse β-globin using specific primers (Table 1) [20]. The PCR reaction was performed in a 50 μl solution with 1x Taq buffer, 2.0 mM MgCL2, 0.2 mM DNTP’S, 0.50 μM of each primer, 1 U de Taq DNA Polimerase and 0.2 μg of genomic DNA. The amplification conditions were as following: denaturation of DNA template at 94°C for 3 min, followed by 35 cycles at 94°C for 30 s 60°C for 45 s, 72°C for 90 s, and a final extension step at 72°C for 10 min. The amplified fragment of 494 base pairs (bp) was analyzed by electrophoresis in 1.5% (w/v) agarose gels stained with ethidium bromide and visualized under UV light.

**Table 1:** Primer sequences.

Primer Target/Name	Sequence	Amplicon
Mouse β-globin		
MBG_fwd	5’-CCAATCTGCTCACACAGGATAGAGAGGGCAGG-3’	494 bp
MBG_rev	5’-CCTTGAGGCTGTCCAAGTGATTCAGGCCATCG-3’	
HPV16-E6		
HPV16_E6fwd	5’-AAAGCCACTGTGTCCTGA-3’	130 bp
HPV16_E6rev	5’-CTGGGTTTCTCTACGTGTTTC-3’	
HPV16-E2		
HPV16_E2fwd	5’-TTTAGCAGCAACGAAGTATCC-3’	184 bp
HPV16_E2rev	5’-AGTCTCTGTGCAACA ACTTAG-3’	

The presence of integrated HPV was assessed by amplification of HPV16-E6 and HPV16-E2 genes with specific primers (Table 1), which amplify a region of 130 bp and 184 bp, respectively, (adapted from a protocol described by Cañadas et al. and Ribeiro et al [21,22]). The PCR amplification reaction with HPV16-E6 and HPV16-E2 primers was carried in a 50 μl reaction mixture with 1x PCR Buffer, 2.5 mM MgCl2, 0.2 mM DNTP’S, 0.30 μM of each primer, 1 U of Taq DNA polymerase and 0.2 μg of genomic DNA. Thermal cycling was performed as follows: initial denaturation of DNA template at 94°C for 2 min, followed by 35 cycles at 94°C for 1 min, 60°C for 1 min, and 72°C for 1 min and a final extension step at 72°C for 5 min. The amplified fragment was analyzed by electrophoresis in 1.5% (w/v) agarose gels stained with ethidium bromide and visualized under UV light.

The resulting genotypes were compared with the respective phenotypes (Table 2).

**Table 2:** Association between the characteristic phenotype of HPV–associated lesions and genotype of HPV E6/E2 DNA of mice.

Mouse	Phenotype	Genotype
1	NEGATIVE	NEGATIVE
2	NEGATIVE	NEGATIVE
3	NEGATIVE	NEGATIVE
4	NEGATIVE	NEGATIVE
5	POSITIVE	POSITIVE
6	NEGATIVE	NEGATIVE
7	POSITIVE	POSITIVE
8	NEGATIVE	NEGATIVE
9	NEGATIVE	NEGATIVE
10	POSITIVE	POSITIVE
11	POSITIVE	POSITIVE
12	POSITIVE	POSITIVE
13	POSITIVE	POSITIVE
14	POSITIVE	POSITIVE

### Sample collection

Seven hemizygous (+/-) and seven wild-type (-/-) females were sacrificed at 22 to 26 weeks of age. Ear and chest skin samples from each animal (14 samples from transgenic and 12 samples from wild-type animals) were collected into TriPure reagent (Roche Applied Science), macerated, and kept at −80°C until processing. Matched samples were collected into 10% neutral buffered formalin for routine histological processing. Histological sections (2 μm-thick) were stained with haematoxylin and eosin (H&E) for examination on light microscopy. Samples were classified as normal skin, epidermal hyperplasia and epidermal CIS by two independent researchers (CL and RGC), as previously described [19].

### miRNA expression analysis

Extraction of total RNA from samples preserved in TriPure reagent was performed using the High Pure Viral Nucleic Acid kit (Roche, Indianapolis, USA), according to manufacturer’s instructions. RNA quality was assessed using NanoDrop spectrophotometer v3.7 (Thermo Scientific, Wilmington DE, USA).

Mmu-miR-155_002571 and snoRNA-202_001232 were analyzed using two-step real-time PCR protocols with TaqMan MicroRNA Assays (Applied Biosystems, Foster CA, USA). The conversion of miRNA to cDNA was performed using TaqMan MicroRNA Reverse Transcription Kit (Applied Biosystems, Foster CA, USA) in a 15 μL of total volume reaction mix with: 7 μL of a master mix containing 1x RT buffer, 1.0 mM of total dNTPs, 50U MultiScribe Reverse Transcriptase Enzyme and 0.25U of RNase inhibitor; 3 μL of RT primers (Applied Biosystems, Foster CA, USA); and 5μL of RNA sample. The amplification conditions were as follows: 30 min at 15°C, 52 min at 42°C and finally 10 min at 85°C. All reverse transcriptase reactions included two non-template controls using double distilled water to replace template RNA.

qPCRs were performed on a StepOne Real-time PCR System (Applied Biosystems, Foster CA, USA) with a 20 μl final volume: 1× TaqMan Universal PCR Master Mix II (Applied Biosystems, Foster City, California USA); 1x MicroRNA Assay (Applied Biosystems, Foster City, California USA); and 2 μL cDNA from RT snoRNA-202 was used as endogenous control. Thermal cycling conditions were: 10 min at 95°C followed by 45 cycles of 15 s at 95°C and 1 min at 60°C. All reactions included two-template controls using double distilled water to replace template cDNA.

### Statistical Analysis

Data analysis was performed using the computer software IBM SPSS Statistics for Windows (Version 20.0). The t-Student test was used to evaluate statistical differences in the normalized expression of the miR-155. In order to analyze the normalized relative expression (-ΔCt) of the different groups, we considered the results corresponding to a 99% representation of the population (*X̅* ± 2SD).

## Results

### Genotyping/phenotyping and histological analysis

**Figure 1:**
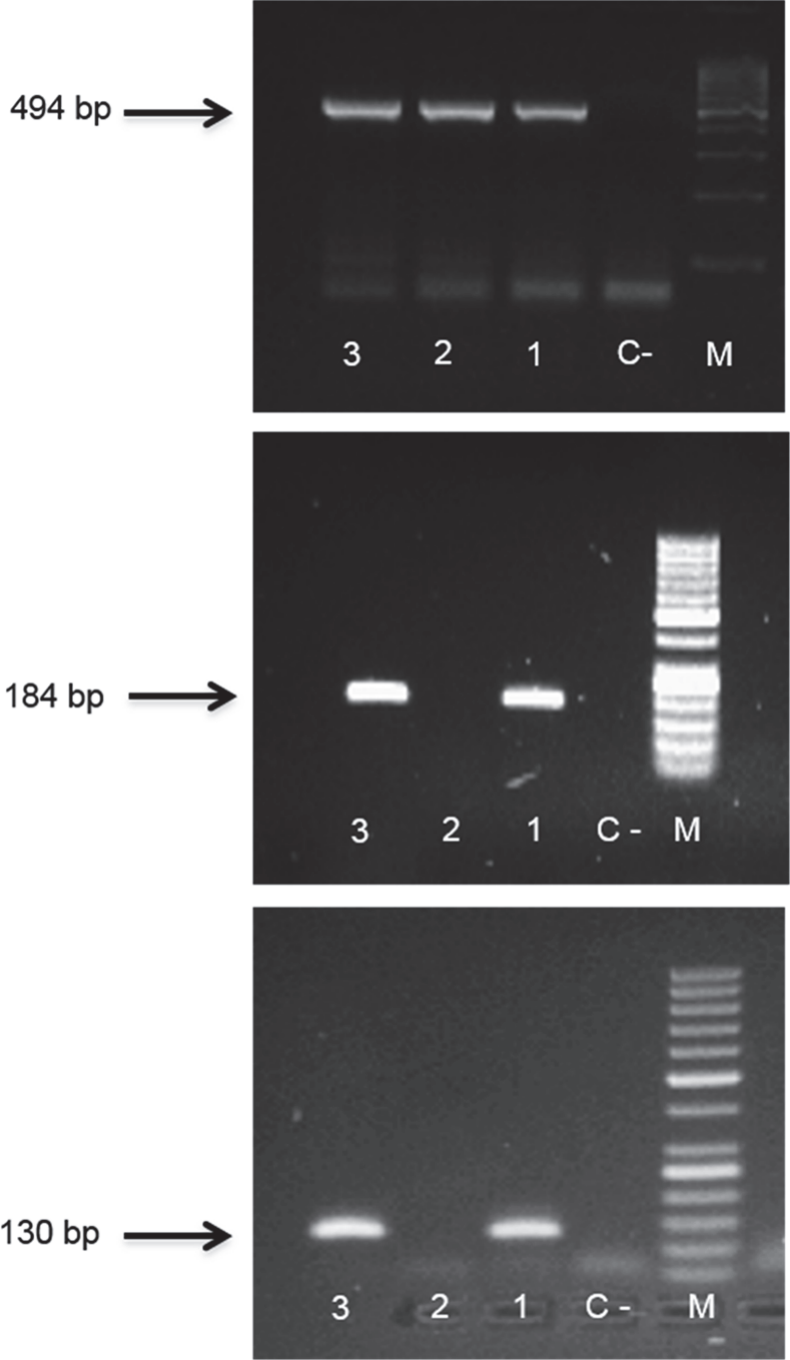
Mice genotyping. The presence of integrated HPV was assessed by amplification of HPV-E2 (b) and HPV-E6 (c) genes by polymerase chain reaction methodology (PCR) in-house. Samples 1 and 3 are HPV+; sample 2 is HPV-. Mouse-β-globin gene was used as endogenous control (a). M: molecular weight size marker: (a)100 bp, (b,c)50 bp; C-: negative control.

**Figure 2:**
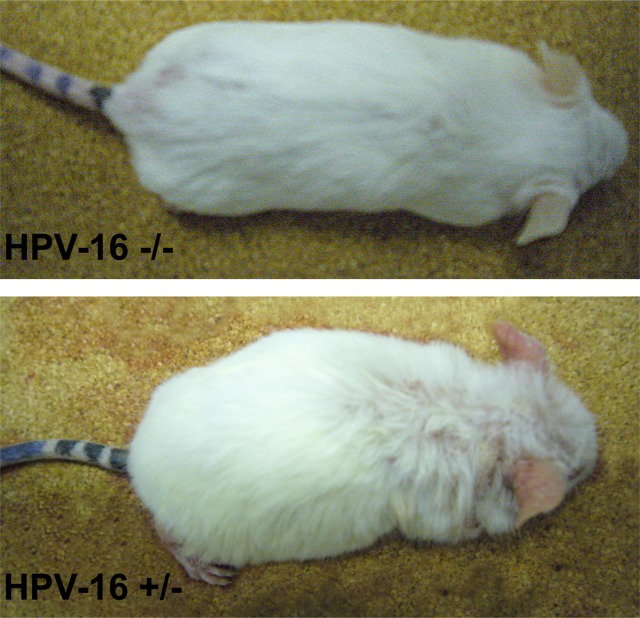
Wild-type (-/-) and K14-HPV16 transgenic (+/-) mice. Transgenic mice show a hunched position, partial thoracic and cephalic alopecia, together with extensive hyperkeratosis and auricular erythema.

**Figure 3:**
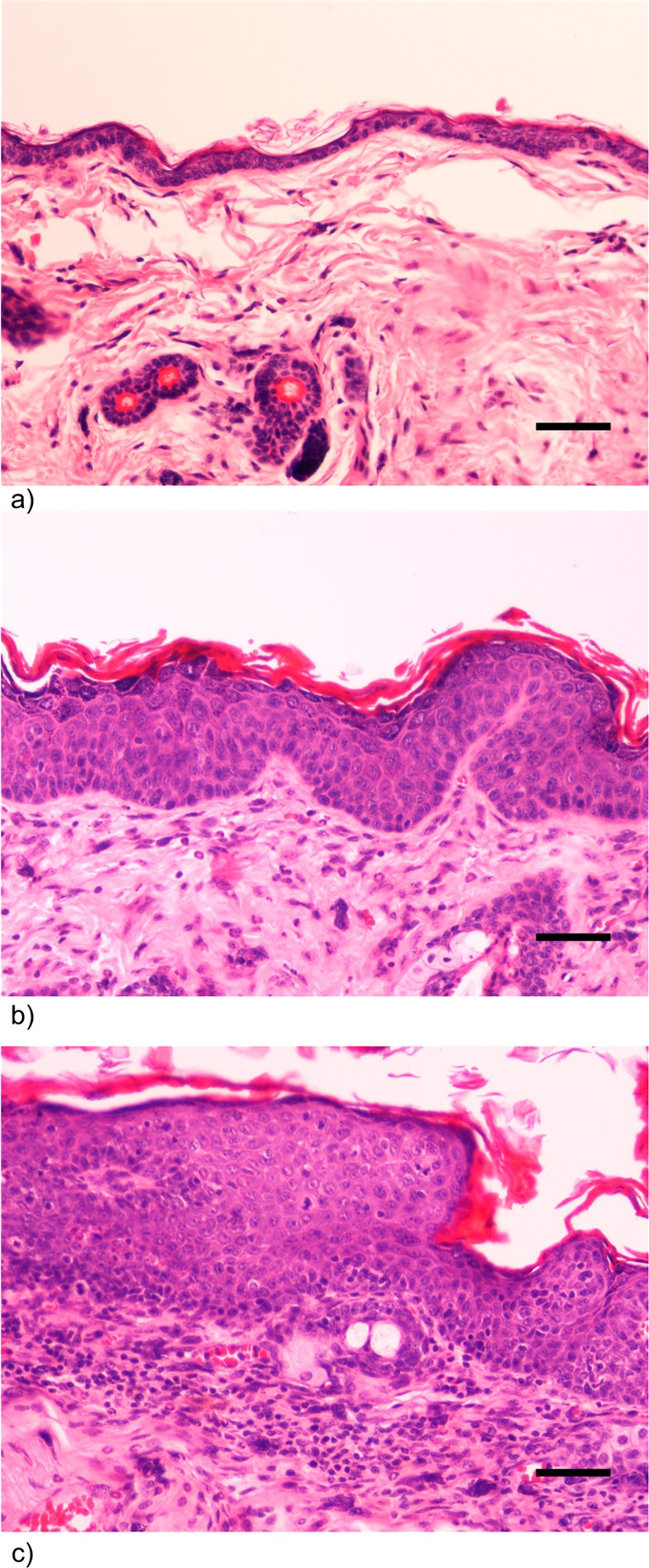
Histology of wild-type and transgenic mice. a) Wild-type (-/-) female FVB/n mouse. Chest skin showing normal histology. H&E, 200x bar = 50μm b) K14-HPV16 transgenic (+/-) female FVB/n mouse. Chest skin showing epidermal hyperplasia and orthokeratotic hyperkeratosis. Note increased number of epidermal strata with conserved orderly squamous differentiation. c) K14-HPV16 transgenic (+/-) female FVB/n mouse. Ear skin showing in situ carcinoma. Note loss of epidermal stratification and progressive differentiation, presence of suprabasal mitotic figures and anisocytosis and abrupt parakeratotic keratinization with hyperkeratosis. The underlying stroma exhibits intense mixed inflammatory cell infiltration and neovascularization.

We observed the presence of HPV16 integration in 7 out of 14 animals (Table 2 and Fig. 1). While wild-type mice did not develop any skin lesion, all mice with integrated HPV DNA demonstrated, phenotypically, various degrees of persistent epidermal squamous hyperplasia and hyperkeratosis, characteristic lesions associated with HPV infection as previously described [19] (Fig. 2). After histologic evaluation, we observed that, in all cases with integrated HPV16, the ear tissues presented CIS, while the chest tissues showed only epidermal hyperplasia, while wild-type mice showed normal skin histology (Fig. 3).

### MiRNA-155 expression profile in tissues from wild-type mice

**Figure 4:**
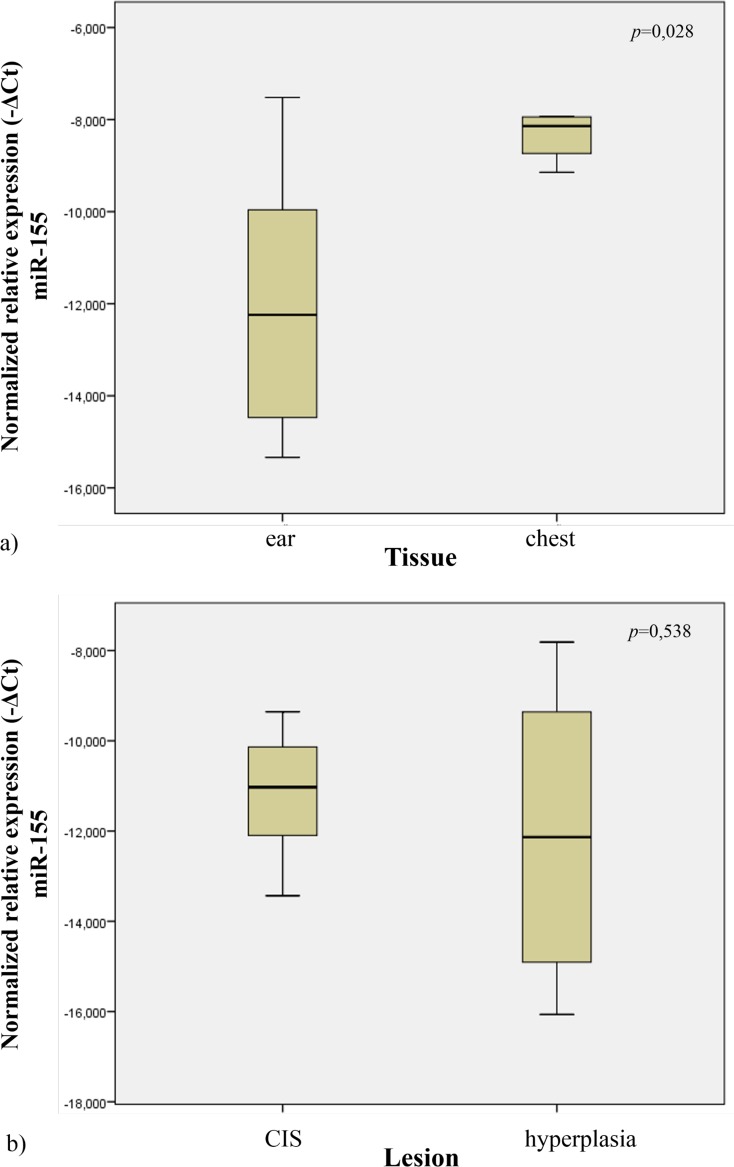
Normalized relative expression of miR-155 in ear and chest normal tissue (a). Normalized expression of miR-155 in different lesions of K14-HPV16 transgenic mice (CIS and hyperplasia) (b).

In order to study the miR-155 expression profile in normal tissues, we quantified its expression in the ear and chest skin samples of the wild-type mice. We observed that the ear tissues had lower expression levels when compared to chest tissues (*p* = 0.028) (Fig. 4A).

### Mir-155 expression profile in tissues of transgenic mice

In order to study miR-155 expression in tissues from transgenic mice, we analyzed the samples from ear and chest skin. We observed no statistical significant difference in miR-155 expression levels between these groups (histologically presenting with CIS *vs* hyperplasia) (*p* = 0.538) (Fig. 4B).

### MiRNA-155 expression profile in normal chest skin versus hyperplastic skin

**Figure 5:**
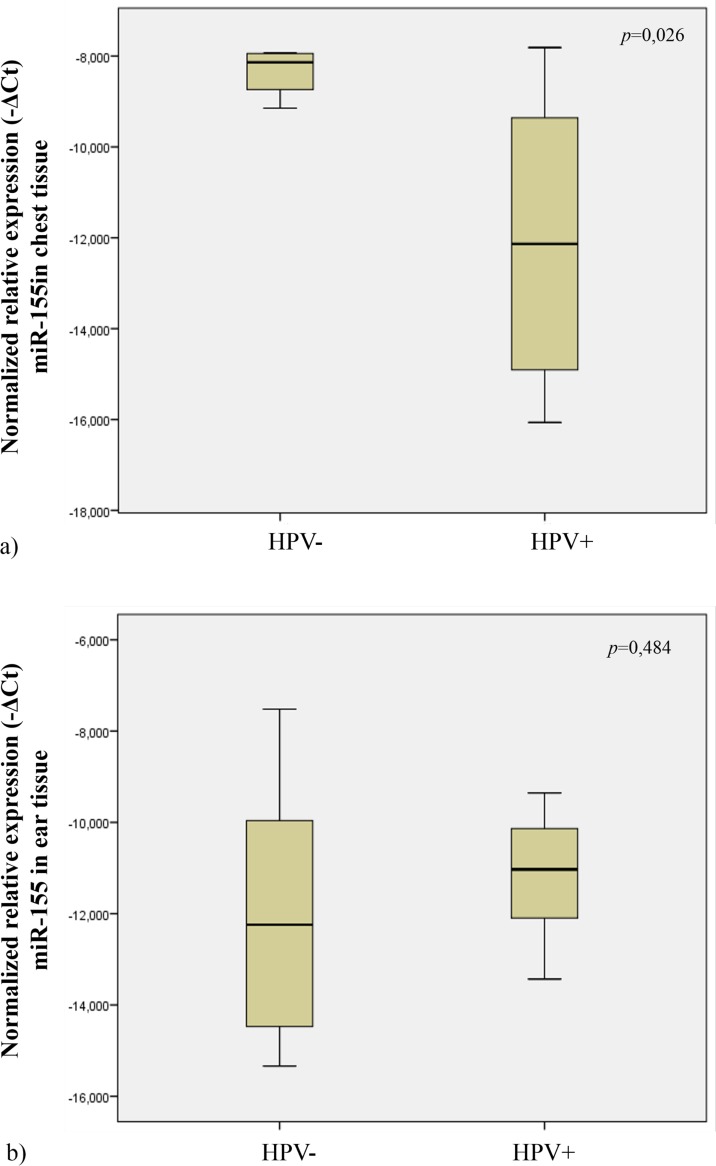
Normalized relative expression of miR-155 in transgenic mice (HPV+) and wild-type mice (HPV-), in chest (a) and ear tissue (b).

In order to explore the possible influence of HPV16 on miR-155 during the early phases of skin carcinogenesis, we studied its expression levels in wild-type (histologically normal) and HPV16-transgenic (hyperplastic) chest skin samples. When comparing wild-type with transgenic chest skin, we observed that, transgenic skin had lower expression levels of miR-155 than normal skin (*p* = 0,026) (Fig. 5A).

### MiRNA-155 expression profile in normal ear skin versus CIS

We also compared the relative miR-155 expression levels on ear tissues of transgenic (showing CIS) and wild-type (showing normal skin histology) mice. Our data showed no statistical significant difference in miR-155 expression levels between these groups (*p* = 0.484) (Fig. 5B).

## Discussion

A large number of different biomarker microRNAs have previously been reported to be connected to cellular transformation. Deregulation of miRNAs is intimately associated with the development and progression of cancer [23,24].

In this context, microRNA profiling studies indicate that deregulation of miR-155 is frequently linked with a wide range of malignancies, including various forms of lymphoma and carcinomas of breast, lung, pancreas, head and neck, and kidney [25–28]. Furthermore, miR-155 is detected during the immune response in activated mature B and T lymphocytes [29], germinal centers B cells [17], and monocytes [16]. BIC/miR-155 knock-out mice exhibited impaired immune response and cytokine production [17], further supporting the vital role of miR-155 in immunology. Much of the current research in the field has implicated miR-155 in promoting oncogenesis. Controversially, recent studies report anti-oncogenic effects of miR-155 [11,12]. Interestingly, these authors found that miR-155 knockdown in myeloid cells facilitated breast cancer development in mice. Some novel concepts that arose from the analysis of these papers were that miR-155 is not only a promoter of some cancers, but may also act to prevent cancer by promoting proper immune function.

It is accepted that HPV infection is the most important factor for transition from normal cervical epithelium to cervical preneoplastic lesions, intraepithelial neoplasia and, subsequently, to invasive cervical cancer [30,31]. The influence of others factors, including the host microenvironment, remains poorly defined. Specifically, in cervical cancer, the most important HPV-associated tumor, no conclusive evidence concerning the relation between HPV and miR-155 expression has been reported. There have been studies on miRNA expression in head and neck cancers reporting miR-155 to be upregulated in oral cancer compared to normal oral tissue [32–34]. However, when compared HPV-positive with HPV-negative squamous cell carcinoma of the head and neck cell lines, this miRNA was downregulated in the presence of HPV-16 DNA [35].

K14-HPV16 transgenic mice are a useful experimental model for studying progressive, multistep HPV-induced carcinogenesis. The FVB/n mouse strain has been shown to be particularly prone to HPV-driven carcinogenesis, as other mouse strains (e.g. Balb/c, C57Bl/6, SSIN/SENCAR) bearing an identical transgene, failed to develop invasive carcinomas [5]. This early observation already highlighted the key role of host factors in the development of HPV-induced cancers. Thus, the characterization of miRNA expression levels in this model animals may be a useful strategy for understanding the mechanisms carcinogenesis associated with HPV.

**Figure 6:**
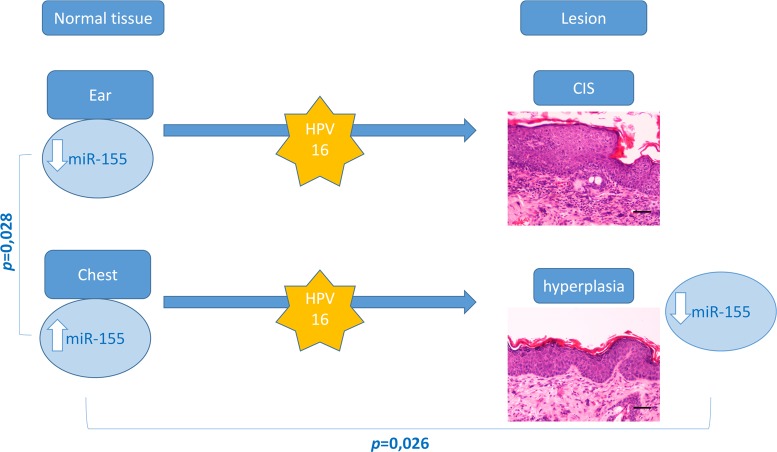
Overview of genotyping, histological and miR-155 profiling results. MiR-155 levels are significantly higher in normal chest skin compared with ear skin samples. Targeted expression of HPV-16 oncogenes to basal keratinocytes leads to multistep skin carcinogenesis–transgenic ear skin samples showed CIS while chest samples showed epidermal hyperplasia. Hyperplastic (chest) skin samples showed a significant miR-155 downregulation compared with matched wild-type samples. No differences were observed between wild-type and transgenic ear samples or between transgenic ear and chest samples.

Cutaneous squamous cell carcinoma develops in multiple locations through well-defined steps. In the present study 22–26 weeks-old transgenic animals (HPV16+/-), showed CIS in all ear skin samples, whereas chest skin samples only showed epidermal hyperplasia. This provided an opportunity to study miR-155 expression levels in different phases HPV16-induced carcinogenesis. Our results (Fig. 6) indicate that, among wild-type mice (HPV-/-), the expression of miR-155 is lower in ear skin tissue compared with chest skin (*p* = 0.028). Also, we observed that hyperplastic chest skin presented lower levels of miR-155 compared with normal chest skin (*p* = 0,026). Based on these results, miR-155 expression levels appear to be a significant microenvironmental factor involved in the development of HPV-associated lesions. Specifically, these results suggest that downregulation of miR-155 may be involved in HPV16-driven early hyperplastic lesions.

In agreement with our findings, a recent study reports that miR-155 acts as a tumor suppressor in human Caski cervical cancer cells (carrying HPV16 DNA). Moreover, it was demonstrated that p53 expression is upregulated by miR-155 overexpression [36]. Recent study indicates that miR-155 overexpression results in decreased cyclin D1 to p21 ratio, suggesting a role in inhibiting cell proliferation [37,38].

Previous reports concluded that interleukin 10 (IL-10) downregulates miR-155 expression post-transcriptionally [39]. Also, women who are genetically programmed to produce high or moderate levels of IL-10 are more likely to develop cervical cancer, compared to individuals genetically predisposed to present low IL-10 production [40]. Our results are in accordance with these reports, suggesting that miR-155 may direct HPV16-induced pathological processes towards hyperplasia rather than malignant transformation.

Other study indicates that miR-155 acts as a positive regulator of interferon gamma (IFN-γ) production [41] and the increase of IFN-γ enhances susceptibility of cervical cancer cells to lysis by tumor-specific cytotoxic T cells [42]. Also, previous reports concluded that miR-155 targets important oncogenes such as B-cell lymphoma 2 (BCL2), which regulates apoptosis [43]. These data may explain the relation between low miR-155 expression levels and cancer development.

Our results showing the decrease of miR-155 expression levels in hyperplastic skin compared with normal chest skin may be explained in the light of these previous reports, including the loss of p53 and the increased of p21 and BCL2, the upregulation of IL-10 and the decrease of IFN-γ levels, promoting an epidermal hyperplasia.

Despite the acknowledged functions of miR-155 in various cancers, further studies are needed to clarify its contribution of theses miRNAs in immunomodulation, and its interaction with cell signaling pathways. Our results suggested that it might be related to the induction of a microenvironment less favorable for HPV-induced carcinogenesis. In conclusion, the data discussed in this article relates a possible anti-oncogenic effect of miR-155. These findings are important in determining the possible role of miR-155 expression in differential tissue predisposition to cancer development.

## Supporting Information

S1 Table MiR-155 expression analysis of ear and chest tissue of transgenic (HPV+) and wild-type (HPV-) mice. (DOC)
