# Correction: A Role for MicroRNA-155 Expression in Microenvironment Associated to HPV-Induced Carcinogenesis in K14-HPV16 Transgenic Mice

**DOI:** 10.1371/journal.pone.0134159

**Published:** 2015-07-21

**Authors:** The PLOS ONE Staff

There is an error in the sixth author’s name. Ana Faustino Carlos Rocha should be two separate authors. The correct names are: Ana Faustino-Rocha and Carlos Lopes.

Ana Faustino-Rocha should be affiliated with 7 Center for the Research and Technology of Agro-Environmental and Biological Sciences (CITAB), University of Trás-os-Montes and Alto Douro, UTAD, Quinta de Prados, 5001–911, Vila Real, Portugal. Carlos Lopes should be affiliated with 4 Experimental Pathology and Therapeutics Group, CI-IPOP, Portuguese Institute of Oncology, Rua Dr. António Bernardino de Almeida, 4200–072 Porto, Portugal.

The correct citation is: Paiva I, Gil da Costa RM, Ribeiro J, Sousa H, Bastos M, Faustino-Rocha A, et al. (2015) A Role for MicroRNA-155 Expression in Microenvironment Associated to HPV-Induced Carcinogenesis in K14-HPV16 Transgenic Mice. PLoS ONE 10(1): e0116868. doi:10.1371/journal.pone.0116868

The publisher apologizes for the error.
