# Supplementary material for: A Role for MicroRNA-155 Expression in Microenvironment Associated to HPV-Induced Carcinogenesis in K14-HPV16 Transgenic Mice
Source: PLoS One. 2015 Jan 27;10(1):e0116868. doi: 10.1371/journal.pone.0116868 (PMC4308071; doi:10.1371/journal.pone.0116868)
Supplement: S1 Table — (DOC) [file pone.0116868.s001.doc]

**Table S1. MiR-155 expression analysis of ear and chest tissue of transgenic (HPV+) and wild-type (HPV-) mice.**

| **Sample** | **Tissue** | **HPV** | **Histology** | **-ΔCt= - Ct (miR-155-snoRNA-202) ^a^** |
| --- | --- | --- | --- | --- |
| **1** | ear | positive | CIS | -12,397 |
| **2** | chest | positive | hyperplasia | -16,065 |
| **3** | ear | positive | CIS | -9,782 |
| **4** | chest | positive | hyperplasia | -7,812 |
| **5** | ear | positive | CIS | -11,794 |
| **6** | chest | positive | hyperplasia | -14,403 |
| **7** | ear | positive | CIS | -11,029 |
| **8** | chest | positive | hyperplasia | -10,602 |
| **9** | ear | positive | CIS | -10,488 |
| **10** | chest | positive | hyperplasia | -8,114 |
| **11** | ear | positive | CIS | -13,432 |
| **12** | chest | positive | hyperplasia | -15,409 |
| **13** | ear | positive | CIS | -9,356 |
| **14** | chest | positive | hyperplasia | -12,134 |
| **15** | ear | negative | normal | -15,337 |
| **16** | ear | negative | normal | -7,519 |
| **17** | chest | negative | normal | -7,953 |
| **18** | ear | negative | normal | -15,048 |
| **19** | chest | negative | normal | -9,146 |
| **20** | ear | negative | normal | -12,318 |
| **21** | ear | negative | normal | -9,225 |
| **22** | ear | negative | normal | -12,164 |
| **23** | ear | negative | normal | -10,699 |
| **24** | chest | negative | normal | -8,325 |
| **25** | ear | negative | normal | -13,896 |
| **26** | chest | negative | normal | -7,934 |

**^a^** ΔCt is the difference of Ct values for the Ct of a miR-155 and Ct of the endogenous control snoRNA-202
